# Targeted Liposomal Drug Delivery to Monocytes and Macrophages

**DOI:** 10.1155/2011/727241

**Published:** 2010-10-26

**Authors:** Ciara Kelly, Caroline Jefferies, Sally-Ann Cryan

**Affiliations:** ^1^School of Pharmacy, Royal College of Surgeons in Ireland, Dublin 2, Ireland; ^2^Department of Molecular & Cellular Therapeutics, Royal College of Surgeons in Ireland, Dublin 2, Ireland

## Abstract

As the role of monocytes and macrophages in a range of diseases is better understood, strategies to target these cell types are of growing importance both scientifically and therapeutically. As particulate carriers, liposomes naturally target cells of the mononuclear phagocytic system (MPS), particularly macrophages. Loading drugs into liposomes can therefore offer an efficient means of drug targeting to MPS cells. Physicochemical properties including size, charge and lipid composition can have a very significant effect on the efficiency with which liposomes target MPS cells. MPS cells express a range of receptors including scavenger receptors, integrins, mannose receptors and Fc-receptors that can be targeted by the addition of ligands to liposome surfaces. These ligands include peptides, antibodies and lectins and have the advantages of increasing target specificity and avoiding the need for cationic lipids to trigger intracellular delivery. The goal for targeting monocytes/macrophages using liposomes includes not only drug delivery but also potentially a role in cell ablation and cell activation for the treatment of conditions including cancer, atherosclerosis, HIV, and chronic inflammation.

## 1. Introduction

Mononuclear phagocytes such as monocytes, macrophages, and dendritic cells are intrinsically involved in innate immunity. As the designation denotes, the chief role of these cells is phagocytosis whereby cells will engulf and destroy apoptotic cells, pathogens, and other targets. This occurs either through employing opsonin receptor-dependent mechanisms via complement- and Fc-receptors, or opsonin receptor-independent mechanisms via lectin-receptors, scavenger receptors, stearylamine receptors or CD14 [1]. 

Due to its pivotal role in inflammation, the mononuclear phagocytic system (MPS) is an important target for drug delivery to treat disease. For certain diseases such as chronic obstructive pulmonary disease (COPD), asthma, atherosclerosis, and cancer [2–4] and for pathogenic infections including tuberculosis [5], human immunodeficiency virus (HIV), and Leishmaniasis [6], the inflammatory process is a key driver of both disease progression as well as pathogenesis. Thus strategies aimed at targeting the MPS are highly attractive. In general however these cells are reputed to be difficult targets [7], particularly where intracellular delivery of the active is required such as for gene delivery [8]. Therefore the development of delivery systems that can target monocytes/macrophages intracellularly is crucial and could potentially open up new treatment paradigms for a range of diseases. 

Liposomes are the most widely investigated delivery system for phagocyte-targeted therapies providing advantages such as low immunogenicity, biocompatibility, cell specificity and drug protection. However, there are also shortcomings such as poor scale-up, cost, short shelf life, and in some cases toxicity and off target effects. Parenterally administered liposomes are naturally cleared by the MPS. Liposomal delivery systems targeting other cell types outside the MPS are modified to evade phagocytosis; for example, “stealth liposomes” include poly-ethylene-glycol (PEG) into their formulations to shield the liposomes from the MPS and increase their circulatory lifespan [9]. Consequently, numerous studies have been carried out to develop formulations that avoid monocyte/macrophage clearance, the corollary of which is that there is now greater knowledge of the mechanisms of binding and uptake that can be harnessed for drug targeting to monocyte/macrophage cells. 

## 2. Monocytes and Macrophages

Cell origin, lineage, and function in the MPS are complex and remain under considerable investigation. In essence, monocytes differentiate from hematopoietic stem cells, specifically granulocyte/macrophage progenitors in the bone marrow and enter the periphery as circulating monocytes. Various microenvironmental cues determine monocyte fate which can lead to differentiation into macrophage and dendritic cells [10]. However monocytes are not simply macrophage and dendritic cell precursors but are also immune effector cells [11]. 

Under inflammatory conditions, circulating monocytes can be recruited to the site of infection or injury, and once there, differentiate. However under steady state conditions, local proliferation maintains resident macrophages in sites such as the lungs and liver. Macrophages (M*∅*s) are central players in the development, progression, and resolution of inflammation [12]. They are polarized following activation into classic (or M1) and alternative (or M2) macrophages [13–15]. M1 macrophages are activated in response to microbial products such as lipopolysaccharide (LPS) or cytokines like interferon-*γ* (IFN-*γ*) and tumour necrosis factor *α* (TNF*α*) and are characterized by a strong propensity to present antigen. In a polarized response, M1 cells are thought to kill intracellular microorganisms and produce abundant proinflammatory cytokines such as TNF-*α*, interleukin (IL)-12, IL-23, and proinflammatory mediators like nitric oxide (NO) and reactive oxygen intermediates (ROI). 

On the other hand, M2 macrophages are promoted by various signals such as IL-4, IL-13, glucocorticoids, IL-10, immune complexes and some pathogen-associated molecular patterns (PAMPs) that elicit different M2 forms (M2a, b and c). They function in inflammation resolution and tissue remodelling. Pathogen Recognition Receptors (PRRs) have evolved to recognise conserved molecular-associated molecular patterns (PAMPS) from pathogens, such as lipopolysaccharide or bacterial DNA motifs. The Toll-like receptors (TLRs) are one such family whose ligands have generated much excitement over the last decade as immunostimulatory adjuvants in vaccine development [16]. Engagement of TLRs by their cognate ligands will activate antigen presenting cells, stimulate cytokine secretion that regulates the adaptive immune response, and promote up regulation of costimulatory molecules in order to improve antigen presentation to T cells. Thus incorporation of TLR ligands or immunomodulatory moieties into liposomes has been a strategy for improving efficacy of both vaccine development and drug targeting [17]. For example, as TLR ligands have been shown to activate macrophages and dendritic cells and enhance antigen-specific T cell responses, then enhanced uptake of PAMP-coated liposomes into these cells would be expected. However, whilst TLR ligands and PAMPs in general can increase liposome uptake, their ability to stimulate and activate macrophages and enhance antigen-specific T cell activation and immune reactivity would suggest that their potential inflammatory properties may be an issue for general use in targeting strategies [18]. In this respect other target receptors such as the scavenger receptors and mannose receptors may prove more appropriate. 

In addition Tumour-Associated Macrophages (TAMs) are an M2-like macrophage population that promote tumour growth via angiogenesis and metastasis, at least in part, by the release of proangiogenic factors including vascular endothelial growth factor (VEGF) and matrix metalloproteinases [19]. Thus targeting strategies aimed at discriminating against M1 and M2 macrophages may be very attractive for cancer chemotherapy in the future [20]. With respect to cancer therapeutics, dendritic cells are major antigen presenting cells that play important roles in cancer detection and elimination through the activation of  T cells, and interest lies in targeting these cells for cancer immunotherapies [21].

## 3. Liposomal Drug Targeting

**Figure 1:**
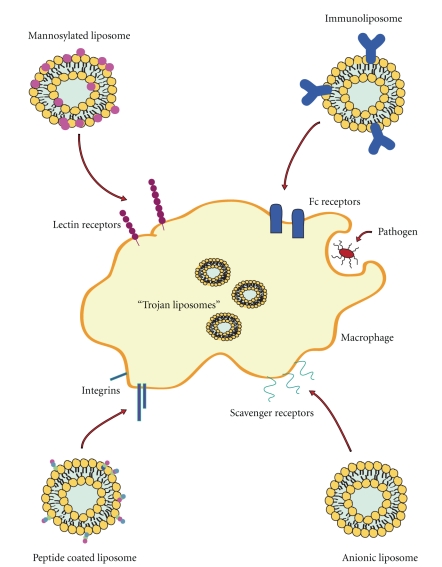
Summary of liposomal targeting strategies to macrophages.

Liposome drug delivery systems harness the physiological role of these cells to provide specific targeting and enhance drug efficacy. Mononuclear phagocytes play major roles in metabolism such as cholesterol and bilirubin metabolism and pathogen clearance [12]. Hence, cell surface receptors are expressed, for example, scavenger receptors that allow the identification and uptake of materials which can be targeted for drug delivery. Targeting of liposomes to monocytes and macrophages can be achieved by modifying lipid composition to control physicochemical properties such as size and charge and by the inclusion of surface ligands including proteins, peptides, antibodies, polysaccharides, glycolipids, glycoproteins, and lectins (Figure 1 and Table 1). 

### 3.1. Physicochemical Properties

Specific liposome properties have been shown to facilitate uptake into monocytes and macrophages and are a simple and effective means of targeting these cells. 

#### 3.1.1. Liposome Size

Recently, a detailed study by Epstein-Barash et al. compared the effect of liposome size and charge on the bioactivity of liposomal bisphosphonates in a wide range of cell types *in vitro* including monocyte/macrophage cell lines (THP-1, J774, and RAW 264 cells) and primary cells (neutrophils, monocytes, kupffer cells, endothelial cells, and smooth muscle cells) and *in vivo * [24]. Liposomes ranged in size from 50 to 800 nm in diameter and were composed of lipids with neutral, positive, or negative charge. It was concluded that small (85 nm) negatively charged liposomes composed of neutral 1,2-distearoyl-sn-glycero-3-phosphocholine (DSPC), anionic distearoyl-phophatidylglycerol (DSPG), and cholesterol at a molar ratio 3 : 1 : 2 were optimum for internalisation by MPS cells while large and positively charged liposomes induced cytokine activation and toxicity [24, 38]. 

While greater uptake of small liposomes (<100 nm) by MPS cells has been reported in the literature [37], many other studies have shown liposome uptake by MPS cells to be improved with increased size [39–41]. Optimal size therefore is likely to be dependent on multiple factors including the target cell and specific properties of the liposome formulation, for example, receptor mediated or nonreceptor mediated uptake. Additionally *in vitro* results often differ from *in vivo* findings [24, 40]. Particularly when administered parentally, liposomes will interact with various circulatory components and are then cleared by hepatocytes *in vivo* [40, 42].

#### 3.1.2. Liposome Charge

Cationic liposomes are associated with efficient cellular delivery of drug cargoes and routinely applied for *in vitro* gene delivery [43]. Electrostatic interactions between positively charged liposomes and the negatively charged cell membranes and cell surface proteoglycans [44] facilitate cell uptake. Unfortunately, cationic liposomes can cause cytotoxicity limiting their safety for clinical use [45]. In RAW264.7 macrophages cationic liposomes containing stearylamine (SA) have previously been shown to induce apoptosis through mitochondrial pathways generating reactive oxygen species (ROS), releasing cytochrome c, caspase-3 and -8 and more recently activating protein kinase C (PKC) *δ* possibly by cell surface proteoglycan interaction [38, 46–48]. Consequently interest for drug delivery has turned to neutral and anionic liposomes. 

**Figure 2:**
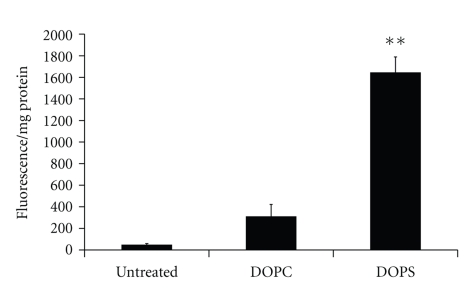
Uptake of neutral (DOPC : Chol 7 : 3) and anionic (DOPS : Chol 7 : 3) liposomes by differentiated THP-1 cells after 2 hours (*n* = 6 ± SEM) *P < .05; **P < .001.

Negatively charged lipids such as phosphatidylserine (PS) and phosphatidylglycerol (PG) are preferentially recognised by macrophages [37]. Studies comparing phosphotidylcholine (PC; neutral) and PS-composed liposomes have established negative liposome formulations to have enhanced macrophage internalisation [49]. Additionally, studies by us to quantify this difference have found a 5.3-fold increase in the association of negatively charged 1,2-dioleoyl-*sn-*glycero-3-phospho-L-serine (DOPS):Cholesterol liposomes with a macrophage cell model, differentiated THP-1 cells, compared to neutral 1,2-dioleoyl-*sn*-glycero-3-phosphocholine (DOPC):Cholesterol liposomes (Figure 2) an effect which was also seen *in vivo* [50]. Negative charge can also be achieved by the incorporation of dicetylphosphate (DCP) [25, 40]. Vyas et al. showed a 3.4-fold increase in rifampicin lung retention in rats when rifampicin was encapsulated in negatively charged DCP, PC, and cholesterol-composed liposomes and a 1.3-fold increase when encapsulated in the corresponding neutral liposomes compared to free drug after aerosol administration [25]. 

The composition of the inner membrane leaflet of eukaryotic cells [1] consists of PS and phosphatidylethanolamine (PE) with an outer layer of PC and sphingomyelin (SM) [51, 52]. In an apoptotic or necrotic event, PS will be exposed on the outer cell surface, and monocytic phagocytosis is induced. It is believed that PS targets scavenger receptors (SRs) on macrophages (Figure 1) but there may also be receptors specific for PS recognition. Moreover PS can activate complement and associate with plasma apolipoproteins such as ApoE promoting phagocytosis by macrophages [53]. There are six classes of SRs with A, B, and D as the most likely participants in liposome recognition [53]. However, not all phagocytes have the same affinity for these anionic lipids. According to Foged et al., PS and PG liposomes were found to have minimal association with human monocyte- and bone marrow-derived dendritic cells [54]. 

In addition PS is a non-bilayer lipid (along with phosphatidylethanolamine; PE) which is frequently used in the development of pH-sensitive and fusogenic liposomes promoting intracellular drug delivery [51]. For instance, liposomes composed of DOPE and PS have been assessed as pH-sensitive carriers of plasmid DNA to RAW 264.7 alveolar macrophages [55]. Recently Andreakos et al. developed a novel amphoteric liposome for the delivery of antisense oligonucleotides to sites of inflammation in experimental arthritis [56]. The novel formulation known as Nov038 is cationic at low pH and anionic at neutral pH, facilitating complexation to nucleic acids and avoiding nonspecific blood interactions, respectively. The group reported targeted delivery to sites of inflammation as well as blood, liver, spleen, and inguinal lymph node mononuclear cells. In addition, Nov038 administration was well tolerated with efficient antisense oligonucleotide delivery *in vivo*.

### 3.2. Ligands

In addition to controlling the physicochemical properties of liposomes to enhance targeting, ligands can be incorporated into liposome formulations to specifically target monocytes, macrophages, and dendritic cells. Using a ligand targeting strategy for liposome drug delivery has the advantages of potentially increasing target specificity and avoiding the need for cationic lipids to trigger intracellular delivery. A multitude of ligands are currently being assessed including peptides, antibodies, proteins, polysaccharides, glycolipids, glycoproteins, and lectins which make use of mononuclear phagocytes characteristic receptor expression and phagocytic innate processes (Figure 1 and Table 1). Here we will briefly look at three of the most commonly studied systems peptide, antibody, and lectin directed delivery. 

#### 3.2.1. Peptides

Cell targeting peptides (CTPs) and cell penetrating peptides (CPPs) have been conjugated to liposomes to improve cell-specific targeting and cell uptake, respectively, to a range of cell types [57]. Peptide sequences such as GGPNLTGRW (GGP-peptide) have been shown to selectively associate with neutrophils and monocytes [58, 59]. GGP-peptide-coated liposomes, with 500 external ligands per liposome, show 30.9 times greater association to monocytes than uncoated liposomes [58]. Arg-Gly-Asp (RGD) peptide has also been incorporated into liposome formulations to target integrin receptors expressed by monocytes [29, 60, 61] (Figure 1). Magnetic RGD-coated liposomes achieved an increase of approximately 15% drug recovery from monocytes and neutrophils compared to uncoated magnetic liposomes [29].

#### 3.2.2. Antibodies

Immunoliposomes are liposomes coupled with antibodies which can be used to target cell-specific antigens. In the case of phagocyte targeting, the use of nonspecific and monoclonal antibodies can lead to liposome opsonisation and uptake by macrophages.* In vivo* liposomes interact with a wide variety of serum proteins including immunoglobulins, apolipoproteins, and complement proteins [42, 53] and may also activate complement leading to enhanced uptake by the MPS. However, protein interaction, complement activation, and opsonisation depend greatly on the physicochemical properties of the liposomes such as size, surface charge, cholesterol content, and lipid composition [42, 53]. For example, some studies have reported complement activation to be greater with increasing liposome size [53] although observed activation has not always been of significance [24]. 

Immunoglobulins (Igs) are recognised by Fc receptors on the surface of phagocytic cells which are involved in phagocytosis as well as antigen presentation [21] (Figure 1). Interest has focused on the Fc*γ*RI receptor as a target which recognises IgG and is expressed by monocytes, macrophages, activated neutrophils, and DCs [21]. Opsonisation is generally Fc-receptor mediated and has previously been shown to significantly enhance liposome uptake by monocytes and macrophages [32]. Opsonisation of non-immunoliposomes by immunoglobulins, for example, IgM and IgG, can also occur *in vivo *leading to enhanced uptake by macrophages [53]. 

Antibodies have been coupled to the surface of liposomes or distally via their Fc-region to liposome-attached PEG [31, 32]. Koning et al. showed increased Kupffer cell uptake with greater antibody surface density [31, 32]. Dendritic cells have been targeted with histidine-tagged antibody fragments attached to a novel chelator lipid, 3(nitrilotriacetic acid)-ditetradecylamine (NTA3-DTDA), incorporated into stealth liposomes via the DC receptors DEC-205 and CD11c [21].

#### 3.2.3. Lectins

Immune cells including alveolar macrophages, peritoneal macrophages, monocyte-derived dendritic cells, and Kupffer cells constitutively express high levels of the mannose receptor (MR). Macrophages and DCs can therefore be targeted via mannosylated nanoparticles (Figure 1). The MR is a C-type lectin 175-kD type I transmembrane protein [62, 63] whose ligands possess a terminal nonreducing sugar such as mannose, glucose, *N*-acetylglucosamine, and fucose [64, 65]. These receptors play numerous roles in immune function including antigenic recognition, endocytosis, and antigen presentation, and are critically involved in homeostatic maintenance, inflammation and immune responses [66, 67]. Hence MR can identify and engulf pathogens such as *Mycobacterium tuberculosis* and *Leishmania donovani *via surface sugar antigens. 

It should be noted that there are a wide variety of lectins with mannose affinity including MR, dendritic cell-specific intercellular adhesion molecule-3 (DC-SIGN) and Endo 180, and many mannose receptor expressing cells but expression and recognition profiles differ between cell types [66]. This is particularly evident during inflammation where expression of MR is altered in DCs [68]. Here we will focus on liposomes designed specifically for macrophage MR recognition (a receptor that is not expressed by circulating monocytes).

**Figure 3:**
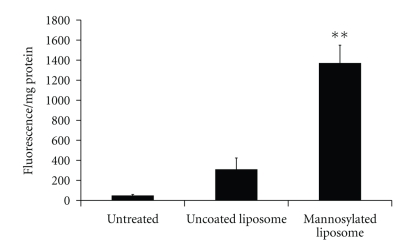
Uptake of uncoated and mannosylated liposomes by macrophage like differentiated THP-1 cells after 2 hours [78]. (*n* = 6 ± SEM) *P < .05; **P < .001.

Mannosylated liposomes have repeatedly been shown to preferentially target macrophages and DCs attaining enhanced cellular uptake both *in vitro* and *in vivo *with better *in vitro/in vivo* correlation than for nonligand containing liposomes [5, 6, 33–36, 41, 49, 66, 69–76]. Mannosylation has been achieved by the incorporation of ligands such as alkyl mannosides [70], Cholesten-5-yloxy-*N*-(4-((1-imino-2-*α*-thioglycosylethyl)amino)butyl)formamide (Mann-C4-Chol) [33, 74, 75, 77], Mann-His-C4-Chol [77], Man_2_DOG [34], 4-aminophenyl-a-D-mannopyranoside [5, 69], and manntriose (Man3)-DPPE [35, 36, 71] into the liposome formulations or by liposome coating with *p*-aminophenyl-*α*-D-mannopyranoside [6]. We have prepared a range of mannosylated liposome, and quantified the increase in cell association with a macrophage-like cell model, differentiated THP-1 cells. Mannosylated liposomes significantly increased liposome association with the macrophages compared to uncoated controls (Figure 3) [78]. 

Over the past decade Hasida and colleagues have led the way in the development of mannosylated liposomes targeted to macrophages and DCs for the delivery of anti-inflammatory agents dexamethasone palmitate [33] and Nuclear factor *κ*-B (NF*κ*B) decoy and anticancer agents CpG oligonucleotides and DNA [79]. Intratracheally administered Man-C4-Chol liposomes were shown to be preferentially taken up by alveolar macrophages which was mediated via MR endocytosis as revealed by inhibition studies. Mannosylation and the extent of this mannosylation significantly improved liposome internalisation by macrophages [72]. The ability of these liposomes to efficiently deliver their load has been the focus of a more recent study in which the use of bubble liposomes and ultrasound in combination with mannosylated liposomes to deliver plasmid DNA to macrophages and dendritic cells was assessed [73]. Significant enhancement of transfection efficiencies was reported using these formulations in comparison to plasmid DNA alone and unmodified liposomes.

## 4. Liposome Drug Delivery for the Treatment of Disease

### 4.1. Infection

A major role of mononuclear phagocytes is the capture and presentation of pathogenic antigens. Certain pathogens are capable of surviving macrophage phagocytosis such as Brucella species [80], HIV [81, 82], and mycobacteria [83]. As a result viruses and bacteria can be harboured and proliferate within these cells. Macrophages can better withstand the cytopathic effects of HIV than T cells [81, 82], while some pathogens such as certain brucella species impair the apoptotic ability of macrophages and monocytes [80], and subsequently survival time of the pathogen-infected cell is extended. As these cells can cross tissue barriers such as the blood brain barrier (BBB), the virus can spread unrestricted [81]. 

The ability of these pathogens to infect, evade the host's phagocytic mechanisms, and replicate creating pathogen reservoirs that can disseminate throughout the body stresses the importance of the development of targeted therapeutics to macrophages and other phagocytic cells. Liposome delivery to these pathogen reservoirs has received some attention [84, 85]. Targeting strategies studied to-date include the use of negatively charged liposomes containing PG [26, 27], sterically stabilized immunoliposomes incorporating surface anti-HLA-DR antibodies [86], tuftsin [87], galactosylated [88], and mannosylated [89] liposomes (Table 1). Overall in these studies, the liposome encapsulation of anti-infectives was generally found to decrease cellular toxicity, modify pharmacokinetics, and improve targeting thereby enhancing the overall efficacy of the anti-infective agents.

### 4.2. Inflammation and Cancer

Mononuclear phagocytes are recruited to sites of injury and cancer, and these sites become areas with a high macrophage presence. As inflammatory cells, macrophages release proinflammatory cytokines such as TNF*α* further increasing inflammation. This process can be utilized in two ways for drug targeting. Firstly, cells can be targeted and activated to bestow tumour suppressive properties for cancer therapy [7]. Secondly, for inflammatory disease, the inflammatory response can be reduced using anti-inflammatory drugs or cell killing to deplete monocyte/macrophage cell populations.

Activation of macrophages is a means of augmenting antitumor immune responses [4] by the induction of proinflammatory mediators such as TNF*α*, IL-8, and nitric oxide (NO) [28]. For instance liposomal delivery of hexadecylphosphocholine [2], JBT3002, a synthetic lipopeptide [3], the tetrapeptide (Thr-Lys-Pro-Arg) tuftsin, and muramyl tripeptide phosphatidylethanolamine (MTP-PE) [28] has been investigated. MTP-PE is a synthetic glycopeptide that can activate monocytes and macrophages promoting tumour regression [28]. A liposomal MTP-PE formulation (L-MTP-PE; mifamurtide) is currently in clinical trials for high risk osteosarcoma. 

Bisphosphonates, for example, clodronate and alendronate, are extensively used in the treatment of osteoporosis but have also shown the ability to induce apoptosis in monocytes and macrophages. Interest lies in their therapeutic potential for inflammatory disorders. To date a range of potential therapies for inflammatory related conditions including nerve injury-associated hyperalgesia [90], endometriosis [91], lung cancer cell metastasis [92], arthritis [93], restinosis [24, 94], and hyperlipidemia [95] have been assessed using liposome-mediated bisphosphonate delivery. Other inducers of macrophage apoptosis have been investigated such as propamidine [96] and locally administered inhibitors such as cycloheximide for atherosclerosis treatment [95].

### 4.3. Cardiovascular Disease

The role of monocytes/ macrophages in the development of atherosclerosis is undisputed [97, 98]. Following endothelial cell damage, monocytes are recruited to the site via the release of chemokines. Following extravasation to the intima, recruited and resident macrophages play a critical role in the development of the atherosclerotic plaque via the scavenging of oxidised LDL and the ultimate differentiation into foam cells which form the atheroscelotic plaque core. The glycoprotein CD36 is central to this process. CD36 is a member of the scavenger receptor class B which is expressed on macrophages/monocytes, platelets, and endothelial cells. Its importance in atherosclerosis has clearly been established through studies in the ApoE-deficient mice, demonstrating that inactivation of CD36 results in substantially reduced lesion size. Therefore targeting of CD36-expressing macrophages in atherosclerotic lesions using a ligand, for example, the growth peptide Hexarelin, can be envisaged to have a dual effect—the delivery of therapeutic agents to the lesion and the neutralisation of LDL uptake. Hexarelin, a member of the hexapeptide growth hormone-releasing peptides (GHRPs), binds to CD36 receptors [99]. 

Investigations into liposome targeting to atherosclerotic lesions have looked at their potential for delivery of contrast agents for diagnostic imaging [100, 101] and anti-inflammatory drugs for therapy development. For instance, Chono and colleagues have investigated liposomal delivery to macrophages as a therapeutic approach to atherosclerosis in several studies [22, 40, 102] using anionic liposomes consisting of egg yolk phosphotidylcholine (PC), cholesterol, and DCP at a molar ratio 7 : 2 : 1 and sized to 70, 200 and 500 nm. *In vitro* uptake by macrophages and foam cells was improved with increasing particle size [22, 40, 102]; however, *in vivo,* optimal aortic delivery in atherogenic mice was achieved using 200 nm liposomes. In addition, various studies have shown significant antiatherosclerotic effects *in vivo* by liposomal delivery of dexamethasone, cyclopentenone prostaglandins, and serum amyloid A (SAA) peptide fragments [22, 30, 103].

### 4.4. Cerebral Ischemia and Stroke

The role of the innate immune system and infiltrating macrophages and resident microglia in cerebral ischemia is currently an area of intense investigation. Inflammation, be it sterile or infection-induced, plays an important part in cerebral ischemic injury. Interestingly CD36 is upregulated in a number of inflammatory and pathological conditions, such as cerebral ischemia and stroke. Both CD36 and TLR2 are upregulated on microglia and infiltrating macrophages under ischemic conditions and triggering either will induce a potent inflammatory response [104, 105]. One study investigated the use of infiltrating macrophages to deliver a systemically administered gene therapy in stroke [106]. Plasmids expressing enhanced green fluorescent protein (EGFP) and fibroblast growth factor-2 (FGF-2) were complexed with cationic liposomes, administered into the femoral vein resulting in expression of EGFP and FGF-2 in infiltrating macrophages and in the cerebral infarction.

### 4.5. Other

There has also been some attention paid to “Trojan monocytes” for drug delivery to the brain [107] as a means of delivering drugs to inaccessible sites (Figure 1). Delivery of drugs to the brain is greatly hampered by the extremely selective permeability of the blood brain barrier (BBB). However, immune cells such as phagocytes can cross this barrier. Therefore by targeting circulating mononuclear cells with drug-loaded liposomes, this natural BBB uptake process can be harnessed for drug delivery.

Previous studies have used RGD-liposomes [29, 60, 61] as well as magnetic liposome formulations [29, 108] for delivery to the brain via monocytes and neutrophils. Afergan et al. prepared PG-composed liposomes for the delivery of the neurotransmitter serotonin [109]. *In vivo* studies showed localisation to the brain to be improved by liposome encapsulation and that the delivered liposomes were intact. FACS analysis of rabbit blood 4 hours posttreatment showed higher uptake of liposomes by monocytes over granulocytes. Uptake was also observed by monocytes and neutrophils *in vivo* and *in vitro* but it was shown that monocytes were the neurodelivery cells by an alendronate monocyte depletion study [109]. More recently Saiyed et al. developed azidothymidine 5′-triphosphate (AZTTP) containing magnetic liposomes as a therapeutic for neuroAIDS [108]. Magnetic nanoparticles (Fe_3_O_4_, magnetite) were encapsulated with AZTTP in neutral liposomes, and transmigration of the liposomes in monocytes was monitored across an *in vitro* BBB model in the presence of a magnet. By magnetic liposome endocytosis, monocytes become magnetic and responded to magnetic fields [29]. The transmigration of magnetic monocytes was significantly increased in the presence of a magnet in comparison to nonmagnetic monocytes.

A study by Matsui et al. examined the potential of peripheral blood monocytes (PBMCs) and human peritoneal macrophages as drug carriers in gastric cancer [36]. Oligomannose-coated liposomes were successfully targeted to monocytes and macrophages showing significantly higher uptake than bare liposomes. These liposome-loaded human monocytes and macrophages were found to accumulate at the disease target site micrometastases and milky spots of the omentum in mice and *ex vivo* in resected human omentum.

## 5. Conclusion

As the role of monocytes and macrophages in a range of diseases including infectious disease, inflammatory diseases, cancer, and atherosclerosis is better understood, strategies to target these cell types are of growing importance both scientifically and therapeutically. Efficient methods of targeting these cells can facilitate efficient drug delivery but also potentially facilitate cell activation and ablation. The properties of liposomes mean they naturally target cells of the MPS, particularly macrophages. This natural targeting capacity can be harnessed for drug delivery. By controlling the liposome physicochemical properties including size, charge, and lipid composition, natural targeting can be further enhanced. A range of ligand-mediated strategies for liposome targeting to MPS cells have been explored including peptide-, antibody-, and lectin-coating to specifically target drug-loaded liposomes to some of the many receptor types expressed on macrophage and monocyte cells.

## Figures and Tables

**Table 1:** Examples of therapeutic applications using monocyte/macrophage-targeted liposomes.

Ligand	Active	Disease	Reference
*Anionic lipids*			
	Dexamethasone	Atherosclerosis	[22]
	SLPI	Inflammatory lung disease	[23]
	Bisphosphonates	Restnosis	[24]
	Rifampicin	Tuberculosis	[25]
	Dideoxycytidine-5′-triphosphate	HIV	[26]
	Clarithromycin	Mycobacterium avium infection	[27]

*Peptides*			
Muramyl tripeptide (MTP)	MTP-phosphotidylethanolamine	Osteosarcoma	[28]
Arg-Gly-Asp (RGD)	Diclofenac sodium (model drug)	Cerebrovascular disease	[29]

*Antibodies*			
Anti-VCAM-1	Prostaglandins	Atherosclerosis	[30]
Anti-CC52	—	Colon Cancer	[31]
Anti-CC531	—	Colon Adenocarcinoma	[32]
Anti-CD11c/DEC-205	tumour antigen (OVA)	Cancer	[21]

*Lectins*			
Mann-C4-Chol	Dexamethasone palmitate	Inflammatory lung disease	[33]
Man_2_DOG	—	—	[34]
Aminophenyl-*α*-D-mannopyranoside	Doxorubicin	Experimental visceral leishmaniasis	[6]
	Ciprofloxacin	Respiratory infection	[5]
Man3-DPPE	OVA		[35]
	—	Gastric cancer	[36]

*Other Ligands*			
Maleylated bovine serum albumin (MBSA)			[25]
*O*-steroly amylopectin (*O*-SAP)			[25]
Fibronectin			[37]
Galactosyl			[37]
